# Stone in the Bladder of a She-ass

**Published:** 1896-08

**Authors:** W. C. Schimmel


Stone in the Bladder of a She-ass. By W. C. Schimmel. On the 13th of November, 1895, a she-ass, nine years old and 1.10 metres high, was received at the Royal Veterinary School

A letter, from a colleague, which accompanied the animal contained the information that she was suffering from stone in the bladder. The animal was in good condition and had a normal appetite, but strained severely on passing urine, so that the vaginal and even the rectal mucous membrane was to a certain extent protruded. With this a small quantity of muddy, slimy urine was emitted, which sputtered out in all directions. On examination the vaginal mucous membrane was found to be swollen and warmer than usual; the opening of the urethra was distended so that a finger could easily pass. The collum vesicæ was not closed; immediately in front of it, partly in the neck of it, a rough, hard stone could be felt; this filled up almost the entire space and could not be moved. On the under side the finger could pass, but on the upper side the stone was held fast in the wall of the bladder. The front end could not be reached. However, we finally succeeded by passing along the lower wall of the vagina; to judge from this the stone must certainly have been 10 cm. long. When the finger-ends were brought in front of the stone, this together with the bladder could be pressed so strongly toward the rear that it became visible in the vaginal opening. The opinion that the stone could be removed without difficulty was not confirmed. At first we tried to extract it with the hand while the animal remained standing. Two fingers could be very easily passed into the bladder, but it was impossible to grasp the stone. With the different kinds of stone-forceps (pincers) we had no better success; the collum vesicæ, although it was quite wide, could not be sufficiently dilated to allow the forceps to pass when they were opened so as to grasp the stone, or when this had been somewhat squeezed, then every effort to extract it was fruitless. The animal was then laid upon its back and another effort made to remove the stone; an incision of considerable size was made in the upper wall of the urethra in order to gain more room for forceps and stone, all, however, without the desired result. In the meanwhile the stone was loosened from its bed so that it could be turned over. Finally we succeeded in pushing off a piece of it; but further efforts to crush it were fruitless. As a result of these manipulations the mucous membrane of the vagina and urethra had become much swollen, and obstructed the use of forceps and fingers and rendered procedure difficult. The constant straining of the animal also impeded our efforts. When after two hours of effort both extraction and crushing seemed impossible, a large incision was made in the collum, so that Bouley's forceps could be used and the stone extracted. A small piece of tissue adhered to it, which gave rise to the belief that the bladder was perforated. This opinion proved to be true. The patient died the following morning. A close examination showed a chronic inflammation of the bladder with thickening of the wall except where the stone had lain; here it had plainly become thinner. The stone had grown into the upper wall, and in one place so deeply that in the efforts to loosen it from its surroundings the wall was perforated before the separation of the stone could be effected. Through this opening urine, gravel, etc., had found access to the abdominal cavity and set up peritonitis. The right kidney was much larger than the left; the capsule could only be stripped from it with great difficulty, which pointed to an interstitial affection. A part, however, was normal, and could still perform its function properly. The left kidney was small; on cutting it open the cavity was found greatly enlarged, and in consequence the medullary substance was absent. Only a part of the cortical substance was present; it was somewhat hydronephrotic. The part of the stone which was removed unbroken had a length of 6 cm. and a circumference of 13 cm. Its weight together with the small pieces which were collected amounted to 127 grammes; about one-third was broken off, so that the almost cylindrical stone must have been 9 cm. long and may have weighed 190 grammes. It was rough, porous on the upper side, dark brown in color, and seemed to have been formed by the aggregation of many small stones. A formation in layers was not noticeable. It belonged to the so-called *sedimentary stones*, as Fürstenberg and Röll have designated them. On the upper surface a part was smooth and still covered with mucous membrane; here it had been firmly attached to the upper wall of the bladder. The stone contained $CO_2$, Ca, Na, HCl, $H_2CO_4$, and $H_3PO_4$; it consisted for the most part of calcium carbonate, besides calcium phosphate and oxalate. According to Röll (*Pathology and Therapeutics of the Domestic Animals*, vol. i. p. 347), sedimentary stones have a form which correspond to that of the bladder, arched below and horizontal above. Our stone differed notably from this, inasmuch as it was almost cylindrical. The lack of a layer formation, however, brings it in this class and not under the white or yellowish-white stones, which develop more slowly, are more solid, and have either compact layers, like the white stones, or alternating layers of a darker and a softer lighter shade, as in the yellowish-white stones — *Tijdschrift voor Veeartsenijkunde*, January, 1896.

